# Divergent Roles of CYP26B1 and Endogenous Retinoic Acid in Mouse Fetal Gonads

**DOI:** 10.3390/biom9100536

**Published:** 2019-09-26

**Authors:** Laura Bellutti, Emilie Abby, Sophie Tourpin, Sébastien Messiaen, Delphine Moison, Emilie Trautmann, Marie-Justine Guerquin, Virginie Rouiller-Fabre, René Habert, Gabriel Livera

**Affiliations:** Laboratoire de Développement des Gonades, UMR Stabilité Génétique, Cellules Souches et Radiations, Université de Paris, Université Paris-Saclay, CEA/DRF/iRCM/SDRR/LDG, 18 route du Panorama, F-92265 Fontenay aux Roses, France

**Keywords:** meiotic entry, retinoic acid, CYP26-enzymes, fetal gonad, electroporation

## Abstract

In female mammals, germ cells enter meiosis in the fetal ovaries, while in males, meiosis is prevented until postnatal development. Retinoic acid (RA) is considered the main inducer of meiotic entry, as it stimulates *Stra8* which is required for the mitotic/meiotic switch. In fetal testes, the RA-degrading enzyme CYP26B1 prevents meiosis initiation. However, the role of endogenous RA in female meiosis entry has never been demonstrated in vivo. In this study, we demonstrate that some effects of RA in mouse fetal gonads are not recapitulated by the invalidation or up-regulation of CYP26B1. In organ culture of fetal testes, RA stimulates testosterone production and inhibits Sertoli cell proliferation. In the ovaries, short-term inhibition of RA-signaling does not decrease *Stra8* expression. We develop a gain-of-function model to express CYP26A1 or CYP26B1. Only CYP26B1 fully prevents STRA8 induction in female germ cells, confirming its role as part of the meiotic prevention machinery. CYP26A1, a very potent RA degrading enzyme, does not impair the formation of STRA8-positive cells, but decreases *Stra8* transcription. Collectively, our data reveal that CYP26B1 has other activities apart from metabolizing RA in fetal gonads and suggest a role of endogenous RA in amplifying Stra8, rather than being the initial inducer of Stra8. These findings should reactivate the quest to identify meiotic preventing or inducing substances.

## 1. Introduction

Germ cells (GC) are unique in their ability to switch from a mitotic to a meiotic program, which is a crucial feature for gamete production. In mammals, the timing of meiotic entry is sexually dichotomic, taking place during fetal life in the ovaries and during postnatal life in the testes. The different fates of male and female fetal GC are influenced and regulated by the somatic environment, independently of their genetic sex (XX or XY) [1,2,3]. The somatic cells of the developing testes repress meiotic entry, while those of the embryonic ovary license GC to initiate meiosis. *Stimulated by RA gene 8 (Stra8)* is considered as key regulator of meiotic entry. 

In both males and females, meiotic initiation is preceded by the expression of *Stra8*. The crucial role of STRA8 in the mitosis/meiosis transition has been highlighted in vivo by analysis of *Stra8 −/−* mutants, showing a pre-meiotic arrest of GC in fetal ovaries and in postnatal testes [4,5,6,7]. *Stra8* was originally identified in P19 embryonic carcinoma cells as a novel gene inducible by retinoic acid (RA) [8]. In a consistent way, the organotypic culture of fetal mouse testes in the presence of RA or agonists of RA receptors (RAR) induces *Stra8* expression, and thus meiotic initiation [4,9,10]. Conversely, cultures in the presence of antagonists of RAR or inhibitors of RA synthesis prevents meiotic entry in fetal ovaries [4,9]. RA is synthesized in the mesonephroi of both sexes during fetal life [4,9,11], and is believed to diffuse in the adjacent gonad. The presence of a RA-synthesizing enzyme has also been reported in embryonic gonads [12]. It has thus been proposed that RA induces *Stra8* and meiosis in the fetal ovary. In parallel, it was found that the RA-degrading enzyme cytochrome P450, family 26, subfamily b, polypeptide 1 (CYP26B1) is expressed specifically in the Sertoli cells of fetal testes. Culturing of fetal testes with the cytochrome P450 inhibitor ketoconazole induces aberrant expression of *Stra8* and meiotic markers [4,9]. The role of CYP26B1 in preventing meiotic entry was confirmed in vivo by the observation of GC initiating meiosis in fetal *Cyp26b1 −/−* mutant testes [9,13,14,15]. Altogether, these findings suggest that RA plays a leading role in the GC decision to enter meiosis. 

Despite the fact that STRA8 and CYP26B1 have mandatory roles in inducing and preventing meiosis, respectively, the evidence that endogenous RA directly controls the mitosis/meiosis transition is still lacking. In this line, it is puzzling that RA signaling is exclusive to metazoans and *Stra8* is a much more recent innovation of vertebrates (Ensembl ENSGT00390000017181), though it is absent in many fish species [16], while meiosis is universal among eukaryotes. In the human fetal ovary CYP26 inhibitor does not stimulate *STRA8* [17] and RA induces a mild stimulation of meiosis (10% after 14 days, [18]). In organ cultures of rat and human fetal testes, RA also poorly induces meiosis and triggers a massive GC apoptosis [19,20,21,22]. It may be considered that the meiotic entry in RA-treated testes is masked by GC loss, as in mice testes RA induces an aberrant meiotic entry that is rapidly accompanied by GC death [10,14]. Albeit in the mouse fetal testes several studies have observed meiotic entry following RA stimulation, some authors did not observe GC meiotic commitment in 11.5 dpc fetal testes cultured with RA [23]. In fetal ovaries cultured without mesonephros, GC enter meiosis, leading to questions about the source of RA [2]. Lastly, in female embryos lacking two RA synthesizing enzymes, *Raldh2−*/*−*; *Raldh3*−/−, *Stra8* is expressed and the GC enter meiosis [24]. Altogether, most studies confirm that exogenous RA is able to stimulate the meiotic entry decision in fetal GC in various species, but the heterogeneity of the observations suggests the need for caution regarding the exact role of endogenous RA in developing gonads.

Although the somatic environment is determinant for GC entry into meiosis, the effect of RA on murine somatic cells has been poorly studied. In embryos, all three RAR (RARalpha, RARbeta, RARgamma) are expressed and their expression is retrieved in both ovaries and testes [4,25,26]; nevertheless, the exact pattern of RAR expression in mouse fetal gonads has never been documented. In this study, we identified a new role of RA in Sertoli cell proliferation in mice, which is not retrieved in *Cyp26b1 −/−* mutants. We thus questioned the role of CYP26B1 as RA-depriving factor in fetal gonads and developed an original technique to ectopically express RA-degrading enzymes, CYP26B1 or CYP26A1, in fetal ovaries. Interestingly, only CYP26B1 reduced the number of STRA8-positive GC, while both enzymes were active in RA signaling.

## 2. Materials and Methods 

### 2.1. Animals

All animal studies were conducted in accordance with the guidelines for the care and use of laboratory animals of the French Ministry of Agriculture (France), and according to protocols approved by the University of Queensland Animal Ethics Committee (Australia) (No. D92-032-02). The mice were housed under controlled photoperiod conditions (lights on from 08:00 to 20:00) and were supplied with commercial food and tap water ad libitum. Embryos were collected from dated matings. Males were caged with females overnight, and the presence of vaginal plug was examined the following morning. The following midday was defined as 0.5 dpc. The mice were sacrificed by cervical dislocation and fetuses were removed from the uterine horns before gonad isolation under a binocular microscope.

The mice used in this study were NMRI (Naval Maritime Research Institute), *Oct4*-GFP, RARA, and CYP26B1 mutant mice. The *Oct4*-GFP mice have previously been described, and were used in cell sorting experiments [27,28,29]. RARA mice have also previously been described [30] and were generously provided by Pr. P. Chambon.

### 2.2. Plasmids

Several plasmids were used for electroporation of the fetal gonads: pCAG H2B-RFP (gift from Dr S. Tajbakhsh); pCIG (IRES-GFP); pCMV5-CYP26B1 (gift from Dr HajimaTakeuchi) coding for the mouse protein CYP26B1, and pSG5-CYP26A1 (Addgene) coding for the mouse protein CYP26A1. Plasmids were extracted from recombinant E.Coli cultures, using Plasmid DNA Maxiprep Kits (Thermo Fisher, Villebon sur Yvette, France), according to the manufacturer’s instructions.

Starting from pSG5-CYP26A1 and pCMV5-CYP26B1 plasmids, *Cyp26a1* and *Cyp26b1* sequences were subcloned into pCIG plasmids. Clonage was achieved through the CIGEX platform (CEA, Fontenay-aux-Roses, France).

### 2.3. Cell Lines and Plasmid Transfection

Human epithelial kidney cells (HEK-293, DSMZ, Braunschweig, Germany) were cultivated in high-glucose DMEM (Gibco, Villebon sur Yvette, France) containing 10% FBS (Gibco, Villebon sur Yvette, France). Plasmids were transfected into cells using Lipofectamine 2000 (Invitrogen, Villebon sur Yvette, France) according to the manufacturer’s instructions. Cells were cultured for 36 h before collection for RT-qPCR analysis. After 24 h of culturing, the medium was changed and DMSO (control) or 10–7M RA was added to the medium for the analysis of RA-target genes expression. After culture, cells were collected in RLT for RT-qPCR analysis. 

### 2.4. Electroporation of Fetal Gonads

Glass capillaries (Harvard Apparatus, Les Ulis, France) were heated and pulled under gravity with a vertical pipette puller (Model 720, David Kopf Instruments, Tujunga, CA). The appropriate sharpness was about 0.05–0.1 mm. Embryos were collected at 12.2 dpc and placed into PBS (Phosphate Buffered Salin) with the amniotic sac intact and the placenta attached. The amniotic sac was opened carefully to inject 5 µL of plasmid solution into the left ventricle of the heart of the embryos using a glass capillary. The injected solution contained plasmids diluted at 300 ng/µL in PBS 1X and Trypan blue (1/10). Trypan blue allowed us to visualize the injected solution and to follow its circulation through the embryos. The solution was injected slowly, following the beating of the heart. During injection, the embryos became blue. The hearts were still beating after injection; embryos with non-beating hearts were excluded from the study. After injection, the embryos were incubated for 30 min in pre-warmed PBS at 37 °C and 5% CO_2_ to allow the plasmid solution to join the gonads’ vascularization. 

After incubation, the whole embryos were blue colored. Both gonads were collected with attached mesonephros. One gonad was directly collected and placed into culture medium (DMEM, 10% FBS). The other one was held between a pair of electrodes for electroporation; electric pulses were applied two times (forward and reverse directions) using an electroporator (NEPA21,Sonidel, Dublin, Irlande ) with the following conditions: 30 V for 35 ms at 50 ms intervals per pulse, and 5 pulses with a decrease of 10% between two consecutive pulses. 

The two gonads with their mesonephroi (the non-electroporated and the electroporated one) were cultured for 48 h. After culturing, the gonads were observed with an inverted microscope (Zeiss, axiovert 200) to visualize the RFP and to assess the percentage of electroporated gonad surface. Of note, pCAG H2B-RFP was always co-injected with pCIG, pCMV5-Cyp26b1 or pSG5-Cyp26a1 to verify the efficacy of electroporation. Next, the gonads were used for immunohistochemical or RT-qPCR analysis. For RT-qPCR analysis, three electroporated or non-electroporated gonads were pooled together. 

### 2.5. Organotypic Culture

Organotypic culture of fetal gonads was performed as previously described [31]. Briefly, fetal gonads were placed on a single Millicell standing insert (HA, pore size: 0.45 μm; diameter: 12 mm, Merck Millipore, Billerica, MA, USA). The inserts bearing the explants were floated in 320 μL culture medium (DMEM/HamF12, 1:1) in 24 wells in tissue culture plates, and incubated at 37 °C, 5% CO_2_. For long cultures, the medium was changed every 24 h. For RA treatments of electroporated gonads, exogenous RA was diluted in DMSO and added to culture medium to obtain a final concentration of 10–7 M. The 12.2 dpc fetal ovaries were cultured for 6 h or 24 h with or without RA at 10–7 M, or the pan-RAR inverse agonist BMS-493 at 10–6 M. WT or *Rara −/−* mutant fetal testes were cultured at 11.5 dpc or 13.5 dpc for 36 h or 48 h, respectively. RA was added to the culture medium at 10–6 M. Talarozole was used at 1 µM (Sigma-Aldrich, Saint Quentin Fallavier, France).

### 2.6. Measure of Testosterone Production

The 12.5 dpc fetal testes were cultured for three days in the presence or absence of 10–6 M RA. During the last 3 h of culturing, 100 ng/mL of luteinizing hormone (LH) was added to the medium. The testosterone secreted into the medium was measured daily as previously described [22,32]. At day three, testosterone secretion was measured before and after LH treatment. Briefly, the culture medium was incubated with anti-testosterone antibody (gift from Dr. Meusy-Dessolle). Then, ^3^[H]testosterone tracer was incubated for 2 h at 4 °C. The bound hormone was counted in a scintillation solution.

### 2.7. Measure of Sertoli Cell Proliferation

The percentage of proliferative AMH-positive Sertoli cells was evaluated by BrdU (5-bromo-2’-deoxyuridine) incorporation, using the Cell Proliferation Kit (GE Healthcare, Buckinghamshire, UK) according to the manufacturer’s recommendations. BrdU (1%) was added at the end of the culture period, or injected into pregnant mice for 3 h before tissue fixation. Next, three to five randomly selected sections were stained for 1 h with anti-BrdU antibody. Peroxidase activity was detected by DAB. The BrdU incorporation index was determined by the counting of at least 500 stained and unstained Sertoli cells. 

### 2.8. RNA Extraction and RT-qPCR

Total RNA was extracted using the RNeasy minikit (QIAGEN, Valencia, CA, USA). cDNA was obtained by reverse transcription using the high capacity kit (Applied Biosystems, Foster City, CA, USA) according to the manufacturer’s instructions. The 7900HT fast real-time PCR system (Applied Biosystems, Foster City, CA, USA) and SYBR-green labelling were used for quantitative RT-PCR (RT-qPCR). The Comparative Ct method was used to determine the relative quantities of mRNA, using *β-Actin* or *Ddx4* (GC-specific marker) mRNA as the endogenous reporter. The results are presented as a percentage of the control (i.e., expression of the control condition being defined as 100%) or as a percentage of the max (i.e., expression of the maximal condition being defined as 100%). Each RNA sample was analyzed in duplicate. All primers were used at a final concentration of 400 nM. Sequences of oligonucleotides used are listed in Table 1.

### 2.9. Purification of Fetal Germ and Somatic Cells

The 13.5 dpc fetal testes and ovaries were collected from *Oct4*-GFP transgene embryos. The gonads were devoid of mesonephros and enzymatically digested as previously described [2,18]. Briefly, 5 min Trypsin (Trypsin/EDTA solution, Sigma-Aldrich, Saint Quentin Fallavier, France) digestion was followed by incubation with 2 mg/mL collagenase and 0.02 mg/mL DNase I. 

*Oct4* specific expression in GC allowed the purification of GC from somatic cells. For purification of Sertoli cells we used α6-integrin staining: the primary antibody, R-phycoerythrin conjugated monoclonal anti-CD49f (BD Bioscience, Le Pont-de-Claix, France), was diluted 1/100 in 2% BSA/PBS (*w*/*v*). HO33258 (0.5 mg/mL-Sigma-Aldrich, Saint Quentin Fallavier, France) was added to exclude dead cells. Cell sorting was performed using ARIA (BD Bioscience). Germ and somatic cells were sorted in RLT buffer (QIAGEN, Valencia, CA, USA) for RT-qPCR analysis. 

### 2.10. Histology and Immunohistochemistry

The gonads were fixed in 4% formaldehyde. The fixed gonads were dehydrated, embedded in paraffin, and cut into 5 μm thick sections. Sections were mounted on slides, dewaxed and rehydrated. For histological analysis, sections were stained with hematoxylin and eosin. 

For immunohistochemical staining, sections were boiled for 20 min in citrate buffer after dewaxing and rehydration. Endogenous peroxidase activity was blocked by incubating sections for 15 min with 3% hydrogen peroxide. Sections were then blocked for 30 min with 2% horse serum and incubated overnight at 4 °C with primary antibody (see Table 2 for antibodies). After three washes in PBS, the slides were incubated with peroxidase conjugated secondary antibodies (ImmPRESSTM reagent kit, Vector Laboratories, Eurobio, Les Ulis, France) for 30 min at room temperature. Antibodies were revealed with DAB (DAB substrate reagent kit, Vector Laboratories) or VIP Vector (VIP substrate reagent kit, Vector Laboratories). 

## 3. Results

### 3.1. Rar Expression in Fetal Gonadal Cells

**Figure 1:**
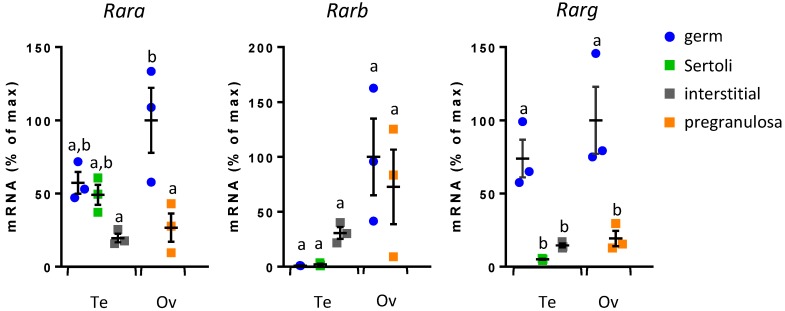
*Rar* are expressed in germ and somatic cells of fetal gonads. Analysis of mRNA expression of the three *Rar* (*Rara*, *Rarb*, and *Rarg*) by RT-qPCR in purified populations of 13.5 dpc *Oct4*-GFP fetal gonads. Testicular (Te) cell populations: germ, interstitial and Sertoli cells; ovarian (Ov) cell populations: germ and pregranulosa cells. Values are normalized to the housekeeping gene *β-Actin*. mRNA expression levels are expressed as percentage of maximum (i.e., mean values for highest conditions are defined as 100%). Different letters indicate significantly different data (multiple comparisons ANOVA). Mean ± SEM, n = 3.

RA binds nuclear RAR, which act as transcription factors to activate RA-target genes. The three *Rar* genes have been reported to be expressed in embryonic germ cells, but their expression in somatic gonadal cells has not been detailed during development. Therefore, we first analyzed mRNA expression profiles of *Rara*, *Rarb,* and *Rarg* in different purified cell populations of 13.5 dpc fetal gonads from both sexes (Figure 1). GC were separated from somatic cells using *Oct4*-GFP mice and male somatic cells were further divided into a6-integrin-positive cells (Sertoli) and -negative cells (interstitium). Cell purity was assessed using markers: *Ddx4*, *Stra8*, *Amh*, and *HSD3b*, respectively specific to germ, pre-meiotic, Sertoli, and interstitial cells (Figure S1). *Rar* are expressed in somatic cells as well as in GC, and in both fetal testes and ovaries. Female GC express the three *Rar*, whereas male GC express only *Rara* and *Rarg*. In Leydig cells the three *Rar* are expressed, whereas Sertoli cells express almost exclusively *Rara* (Figure 1).

### 3.2. RA Increases Mouse Fetal Testosterone Production

**Figure 2:**
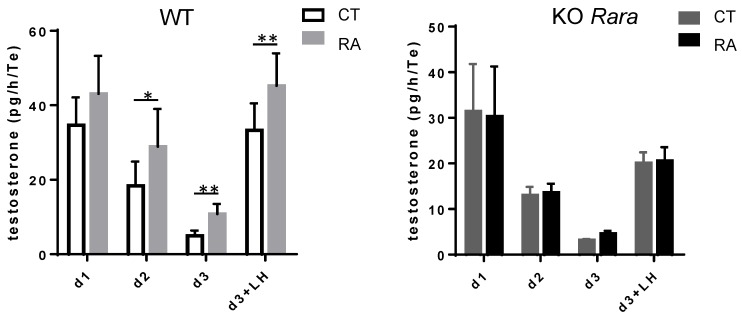
Retinoic acid (RA) increases Leydig cell testosterone production. Testosterone secreted (pg/h/testis) by 12.5 dpc WT (n = 7) and *Rara −/−* (KO *Rara*, n = 5) testes cultured for 1, 2, and 3 days (d1, d2, and d3, respectively) in the presence or not of 10-6M RA (CT and RA respectively); d3 testes are stimulated by LH during the last 3 h of culture (d3 + LH). * *p* < 0.05; ** *p* < 0.01 (parametric *t*-test) Mean ± SEM.

We next asked about the role of RAR in the somatic cells of fetal testes. Testosterone production by fetal Leydig cells is crucial for the proper masculinization of the embryo and has previously been reported to be altered following RA exposure in the developing rat and human testes [22,33]. Therefore, we cultured 12.5 dpc mouse fetal testes in the presence of exogenous RA and measured testosterone production. RA increases the secretion of testosterone starting at day two of culturing (Figure 2). RA stimulation of testosterone production is maintained in LH-stimulated Leydig cells on the third day. In rat fetal testes, RA effects on steroidogenesis are mimicked by an RARalpha agonist [34]. We thus investigated RA effect in *Rara −/−* mutant mice. Of interest, RA does not increase testosterone production in mouse fetal testes from *Rara −/−* mice. 

### 3.3. RA Inhibits Sertoli Cell Proliferation

**Figure 3:**
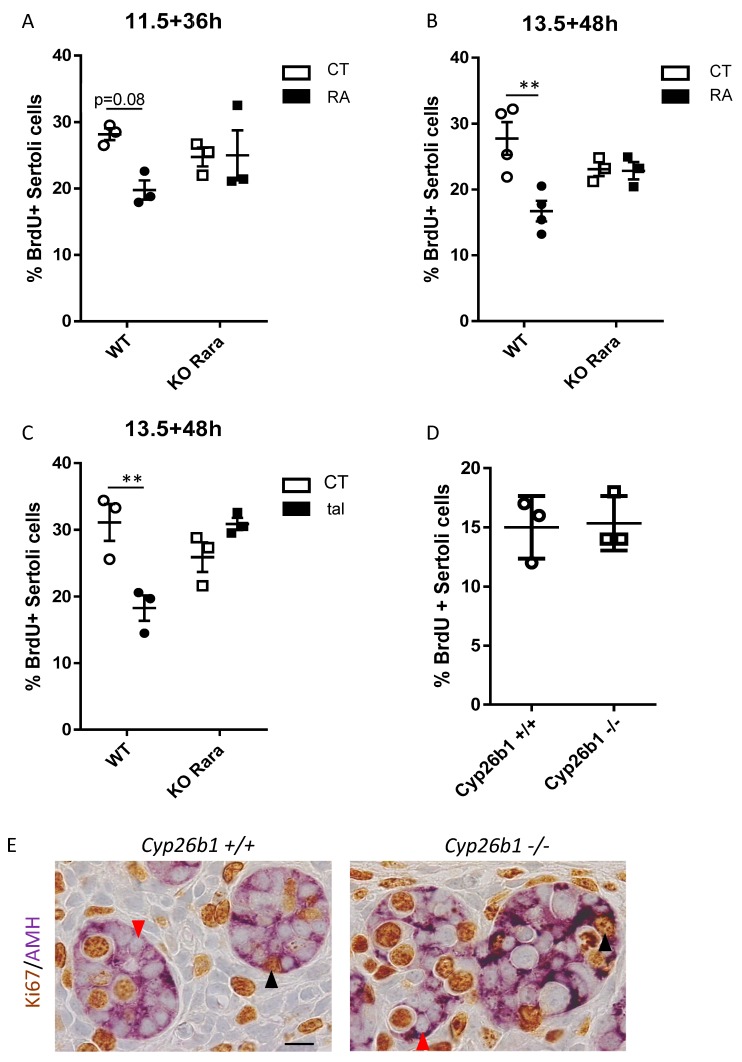
RA inhibits Sertoli cell proliferation. (**A**,**B**) 11.5 and 13.5 dpc WT and *Rara −/−* mutant (KO *Rara*) testes were cultured for 36 h and 48 h, respectively, in the presence or not of 10-6M RA (CT and RA). Percentage of BrdU-positive/AMH-positive Sertoli cells was measured. (**C**) 13.5 dpc WT and *Rara −/−* mutant (KO Rara) testes were cultured for 48 h in the presence or not of 1 µM of Talarazol (CT and tal). (**D**) Percentage of BrdU-positive/AMH-positive Sertoli cells in testes of *Cyp26b1 +/+* and *Cyp26b1 −/−* 13.5 dpc embryos. Mean ± SEM, n = 3. ** *p* < 0.01 (two-way ANOVA). (**E**) Immunohistochemical detection of Ki67 and AMH in sections of 13.5 dpc fetal testes. Red arrows indicate Ki67-negative/AMH-positive cells; black arrows indicate Ki67-positive/AMH-positive cells. Scale bar: 10 µm.

We next analyzed RA’s effect on Sertoli cell proliferation in cultured fetal testes. RA treatment reduced the percentage of BrdU-positive/AMH-positive cells in 13.5 dpc testes after 48 h (Figure 3). A similar inhibition was found in 11.5 dpc testes cultured for 36 h when BrdU incorporation was measured in pre-Sertoli cells located beneath the coelomic epithelium. This suggests that Sertoli cell proliferation is inhibited by RA as early as these cells begin to differentiate. The effect of RA-inhibited Sertoli cell proliferation is abolished in fetal testes from *Rara −/−* mutant mice. Altogether, this indicates that exogenous RA inhibits the proliferation of fetal Sertoli cells via RARalpha.

### 3.4. Cyp26b1 Invalidation Does Not Inhibit Sertoli Cell Proliferation

Establishing that exogenous RA inhibits Sertoli cell proliferation does not imply that endogenous RA has a similar activity. We thus measured Sertoli cell proliferation in the presence of talarozole, a well-known inhibitor of CYP26 RA-degrading enzymes. In 13.5 dpc fetal testes cultured for 48 h with talarozole, Sertoli cell proliferation was inhibited and this effect was absent in *Rara −/−* mutant mice (Figure 3). Thus, endogenous RA indeed inhibits Sertoli cell proliferation via RARalpha. This is also consistent with RARalpha being the sole *Rar* present in Sertoli cells. We thus speculated that CYP26B1 presence in fetal testes should favour Sertoli cell proliferation. To test this hypothesis, we analyzed BrdU incorporation in vivo in *Cyp26b1 −/−* fetal testes. Surprisingly, in these mutants, Sertoli cell proliferation was unchanged compared to wild-type fetal testes (Figure 3). This discrepancy may be due to the difference of models (i.e., constitutive invalidation in vivo vs. punctual drug treatments in vitro). Nonetheless, it casts a first doubt about the exact function of CYP26B1 in mouse embryonic gonads.

### 3.5. Short-Term Inhibition of RA Signaling Does Not Affect Stra8 Expression

**Figure 4:**
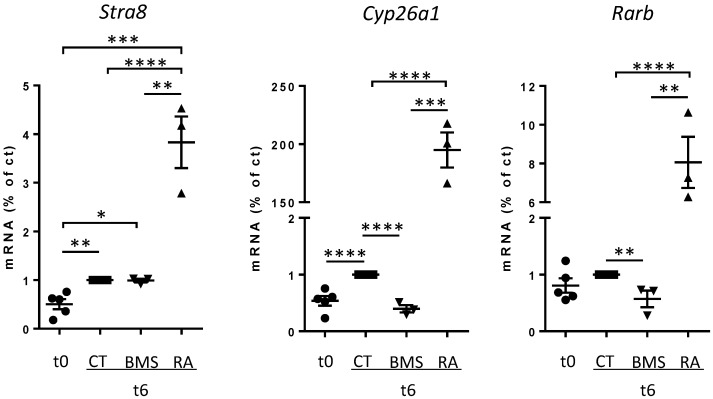
Short-term inhibition of RA signaling does not prevent *Stra8* expression. Fetal ovaries were collected at 12.2 dpc (t0) and cultured for 6 h (t6) in culture medium (CT) or in medium with 10–7 M RA or 10–6 M BMS 493. mRNA expression levels of RA-target genes (*Stra8*, *Cyp26a1,* and *Rarb*) were analyzed. *Stra8* values were normalized to the GC specific marker *Ddx4*; *Cyp26a1* and *Rarb* values were normalized to the housekeeping gene *β-Actin*. RNA levels are expressed as percentage of controls (i.e., t6 CT values are defined as 1) Mean ± SEM, n = 3–5. * *p* < 0.05; ** *p* < 0.01; *** *p* < 0.005; **** *p* < 0.001 unpaired *t*-test.

Currently, CYP26B1 is considered the meiotic preventing factor in fetal testes due to its RA-degrading activity. On the other hand, RA is considered the key inducer of *Stra8* in fetal ovaries. As we observed in the fetal testis an RA role that was not present in *Cyp26b1 −/−* embryos, we also detailed the RA-dependency of *Stra8* in fetal ovaries. Mouse fetal ovaries were collected precisely at 12.2 dpc when *Stra8* starts to be expressed at low levels and were set in organ culture for a short time. After 6 h, the level of *Stra8* spontaneously increased (Figure 4). 

In these conditions, addition of BMS 493, an inverse agonist of the three RAR, does not prevent the increase of *Stra8* nor decrease *Stra8* mRNA, while exogenous RA enhances *Stra8* expression. To verify the efficacy of BMS 493 treatment in organ culture, we measured the expression levels of well-known RA-targets, namely *Cyp26a1* and *Rarb*. As expected, both were significantly decreased in the presence of BMS 493, confirming the efficiency of RA-signaling inhibition (Figure 4). These results may appear odd, as several studies have reported that inhibition of RAR activity diminishes *Stra8* expression in the mouse ovary. However, most have measured *Stra8* expression after long-term treatment (over 24 h). We thus performed similar experiments and after 24 h BMS 493 decreased *Stra8* expression in culture mouse fetal ovaries (Figure S2). These results suggest that endogenous RA amplifies *Stra8* expression but is not mandatory for the initiation of *Stra8* expression.

### 3.6. Ectopic Expression of CYP26B1 or CYP26A1 Differentially Regulates STRA8 in Fetal Ovaries

**Figure 5:**
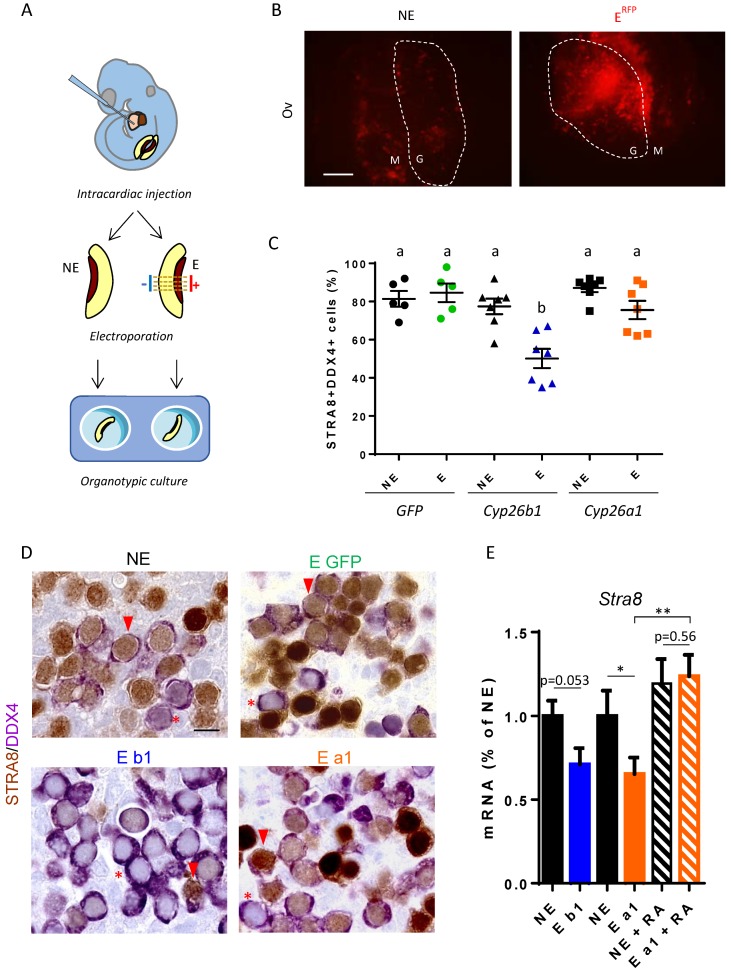
Ectopic expression of CYP26A1 does not reduce the number of STRA8-positive germ cells. (**A**) Schematic representation of the experimental protocol used to electroporate plasmids into 12.2 dpc fetal gonads: Intracardiac injection of plasmids diluted in PBS and trypan blue; after 30 min one gonad was electroporated (E). The contralateral gonad is used as a non-electroporated control (NE). E and NE gonads were cultured for 48 h on inserts (organotypic culture). (**B**) Red Fluorescence Protein (RFP) detection in ovaries (Ov) NE or E with RFP plasmid. White dotted lines encircle gonads (G). Adjacent mesonephros (M) is indicated. Scale bar: 200 µm. (**C**) 12.2 dpc fetal ovaries were non-electroporated (NE) or electroporated with GFP (E GFP, green), CYP26B1 (E b1, blue) or CYP26A1 (E a1, orange). After 48 h of culturing, the percentage of STRA8-positive/DDX4-positive cells is represented. GFP: n = 5; CYP26B1: n = 7; CYP26A1: n = 7. Different letters indicate significantly different data (multiple comparisons ANOVA). (**D**) Immunohistochemical detection of STRA8 and DDX4 in sections of fetal E or NE ovaries. Red stars indicate STRA8-negative/DDX4-positive cells; red arrows indicate STRA8-positive/DDX4-positive cells. Scale bar: 50 µm (**E**) mRNA expression level of *Stra8*. Values are normalized to *Ddx4* (germ cell specific marker). Ovaries were non-electroporated (NE) or electroporated with CYP26B1 (E b1) or CYP26A1 (E a1). The RA treatment of CYP26A1-electroporated ovaries prevented the decrease of *Stra8* expression. * *p* = 0.041; ** *p* = 0.0071 (paired *t*-test between E and contralateral NE ovaries; unpaired *t*-test between ovaries from different embryos) n = 5.

In order to clarify the roles of RA and CYP26B1 in mouse gonads, we developed an original approach to artificially induce CYP26 proteins in fetal gonads. The experimental protocol was based on intracardiac injection of plasmids into 12.2 dpc embryos, followed by their electroporation in fetal gonads. For each embryo, one gonad was electroporated (E), while the other one was used as a non-electroporated (NE) control. Gonads with their attached mesonephroi were then cultured for 48 h (Figure 5A).

First, we validated the experimental procedure by electroporating plasmids coding for Red Fluorescence Protein (RFP) and Green Fluorescence Protein (GFP) in fetal gonads. The macroscopic observation of whole ovaries and testes after 48 h of culturing allowed us to detect red and green fluorescence in the electroporated gonads (Figure 5; Figure S3), in over 20% of the gonad area (Figure S3). This corresponds roughly to the places were electrodes were positioned. RFP immune-detection revealed its presence exclusively in electroporated gonads, albeit in lower abundance (Figure S3). When RFP and GFP plasmids were co-injected, they displayed a near perfect co-localization, indicating incorporation of plasmids in the same area. In all later experiments, RFP plasmids were systematically co-injected with the plasmid of interest to ensure the efficacy of electroporation. Finally, we analyzed the morphology of gonads at the histological level. Electroporated and non-electroporated gonads displayed comparable morphologies (GC presence, cords in testes, and cells initiating meiosis in ovaries), suggesting that the electric pulses did not alter the tissues. 

Second, we compared the activity of CYP26B1 to that of CYP26A1 in fetal ovaries. CYP26A1 is known to be a potent RA-degrading enzyme and its specificity towards retinoids has been well characterized. If both enzymes act solely through RA degradation, we expect their ectopic expression will display identical phenotypes. As a control of the efficiency of the plasmids we transiently expressed CYP26B1 or CYP26A1 in HEK-293 cells, and both reduced the expression of the RA-target gene *Rarb* (Figure S4). This confirms that both CYP26B1 and CYP26A1 indeed have the ability to degrade RA in vitro, as expected. Of note, cell sorting indicated that RA degradation by overexpressing CYP26 also affects untransfected nearby cells, as *Rarb* expression was also inhibited in cells from the same plate that did not express CYP26 (Figure S4). 

In GFP-electroporated fetal ovaries, STRA8 protein was present in about 80% of GC after 48 h of culturing, like in non-electroporated ovaries, indicating that the cells were entering meiosis (Figure 5C). Using this system, the ectopic expression of CYP26B1 reduces the number of STRA8-positive GC by 35%. Of note, STRA8-negative cells are often grouped together, likely corresponding to the electroporated region. Thus, CYP26B1 gain of function in the mouse ovary nicely confirms that CYP26B1 is sufficient to prevent meiotic entry. Surprisingly, ectopic expression of CYP26A1 hardly reduced the number of STRA8-positive cells in the ovary. This evidences that CYP26A1 or CYP26B1 overexpression in mouse developing ovaries leads to different outcomes. Though the number of GC expressing STRA8 appeared unchanged, CYP26A1 reduced the amount of *Stra8* mRNA, indicating that RA does regulate *Stra8* transcription to some extent (Figure 5E). Confirming this, RA addition restored *Stra8* expression in the presence of CYP26A1. A similar antagonist regulation with CYP26A1 and RA occurs in the fetal testes, in which the basal expression level of *Stra8* is very weak compared to that of fetal ovaries (Figure S5). Altogether, this indicates that endogenous RA stimulates *Stra8* transcription and that this effect is abolished by electroporating CYP26A1 in fetal gonads. On the contrary, in gonads electroporated with CYP26B1, *Stra8* expression is fully abrogated in about half of the GC, suggesting that CYP26B1 prevented its induction.

## 4. Discussion

This report provides evidence, for the first time, that the addition of CYP26B1 to the fetal gonad is sufficient to prevent meiotic entry. We also bring forth new data demonstrating that RA addition modifies the behavior of male somatic cells in vitro. Additionally, inhibition of RAR and overexpression of RA degrading enzymes indicate that RA regulates the late onset of *Stra8* expression in the ovary. Altogether, this leads to a possible reconsideration of the roles of CYP26B1 and RA in the mitotic/meiotic switch.

Using an organ culture of mouse fetal testes, we demonstrated that RA increases testosterone production. This RA action on steroidogenesis is opposite to that which we described previously, indicating that RA decreases testosterone production in the developing rat testis, but is similar to that reported in human fetal testes [22,33]. RA effects on steroidogenesis are thus highly variable. Of interest, RAR agonists in rats and *Rara* KO in mice (present study) indicate that in both cases RA acts via RARalpha. However, this does not mean that RA acts directly on Leydig cells. As the three RAR are expressed in fetal Leydig cells, it is surprising that the sole invalidation of RARalpha blunted the RA effect on testosterone production. We thus speculate that this RA-induced increase of steroidogenesis may be mediated by paracrine signals originating from Sertoli cells that only contain RARalpha. That exogenous RA stimulates testosterone secretion in vitro does not mean that RA is a regulator of the fetal testicular steroidogenesis in vivo, especially as the fetal testis is considered to be deprived of RA due the presence of the RA-degrading enzyme CYP26B1. However, in *Cyp26b1 −/−* mutant mice it was recently reported that Leydig cell differentiation is impaired and that steroidogenesis is decreased [35]. Such data provide a first hint that RA addition does not recapitulate *Cyp26b1* invalidation. 

We report similarly that fetal Sertoli cell proliferation is inhibited by RA addition and by chemical inhibition of CYP26 in organ culture. This demonstrates that exogenous and endogenous RA inhibit Sertoli cell multiplication. We also demonstrate that this effect involves RARalpha, as it is the sole RAR present in Sertoli cells, and that *Rara −/−* mutants are refractory to this RA-mediated inhibition of Sertoli cell proliferation. Collectively, these data indicate that Sertoli cells are sensitive to RA. This is unexpected, as these cells express a high level of CYP26B1 that should protect them from RA. We also report that, similarly to Leydig cells, the effect of RA on Sertoli cells is not recapitulated in *Cyp26b1 −/−* embryos. Altogether, one may consider that *Cyp26b1* invalidation is constitutive and may thus have triggered compensatory mechanisms that are not observed when the gonads are stimulated by RA in culture. On the other hand, it may also suggest that RA degradation is not the main activity of CYP26B1 in the fetal gonads. 

To challenge this hypothesis, we artificially expressed CYP26B1 or CYP26A1 in mouse fetal ovaries and observed that they regulated differentially the apparition of pre-meiotic STRA8-positive cells. Only CYP26B1 prevented the apparition of STRA8 in GC. This effect is well correlated to the extent of electroporation and proves that CYP26B1 gain of function in a fetal ovary, just prior to meiotic entry, is sufficient to inhibit the mitotic/meiotic switch. This data is complementary to *Cyp26b1* invalidation in the developing testis that is sufficient to allow the expression of *Stra8* [9]. Altogether, it shows that CYP26B1 is necessary and sufficient and confirms its key function in the machinery that prevents meiotic entry in GC. Surprisingly, CYP26A1 expression in fetal ovaries did not prevent the formation of new STRA8-positive cells. CYP26A1 is an enzyme that has been extensively characterized and proven highly specific and efficient in the degradation of RA [36]. Actually, its RA degrading activity is tenfold higher than that of CYP26B1. Thus, we consider two hypotheses: first, CYP26A1 was inefficient to degrade RA in our model. This is unlikely, as we ensured the efficiency of the *Cyp26a1* sequence used and proved that it efficiently inhibited RA-target genes in HEK293 cells. Additionally, though the number of STRA8-positive cells was not modified, the abundance of the *Stra8*-transcript was decreased in the CYP26A1-electroporated ovary, indicating that the plasmid was able to display some activity. The second hypothesis would consider that if CYP26A1 functions as a potent RA-degrading factor, then CYP26B1 should have an additional activity independent of RA. This would equally fit with the discrepancies between RA-observed effects and those of *Cyp26b1* invalidation in male somatic cells. While such a view is challenging, it should be noted that CYP26B1 and CYP26A1 only share 42% amino acid sequence identity [37] and that, based on this homology, CYP26B1 activity has mostly been tested against retinoids. This opens the possibility that CYP26B1 may have other substrates. Such a speculation is strengthened by the likely differences in active sites of the two enzymes, revealed by their different responses to various selective inhibitors [38].

Nonetheless, many studies, including ours, have reported that RA enhances *Stra8* expression in GC, and this is consistent with *Cyp26a1* gene transfer experiments in the embryonic ovary reported here. We thus propose that such an RA stimulation of *Stra8* gene expression is only possible when a first signal has unlocked Stra8. This signal is likely dependent on CYP26B1. In such a model, *Stra8* would first be induced by an RA-independent signal which remains to be identified and is inhibited by CYP26B1. This is compatible with our data, demonstrating that in an embryonic ovary, when *Stra8* starts to be expressed, this early expression is insensitive to RAR blocking drugs. Later, RA would reach the STRA8–positive GC and then possibly amplify the transcription of *Stra8*. In agreement with this model, it was recently reported that RAR are dispensable for meiosis initiation in mouse fetal ovaries [39]. Following this line, the actual regulator of the meiotic switch, which was previously termed the ‘meiotic preventing substance’, remains to be identified. Of interest, based on co-culture and conditioned media, we previously proposed that such a factor should be a small diffusible molecule [2]. Such a description of course does not fit with the CYP26B1 enzyme and may only potentially apply to one of its products.

## 5. Conclusions

Our study revealed discrepancies between CYP26B1′s supposed role and the RA effects. These suggest that this enzyme, which we confirmed is key to orchestrating the mitotic/meiotic switch, may have additional activity apart from degrading RA. This implies that RA may not be the initial signal triggering Stra8 and meiosis. 

## Figures and Tables

**Table 1:** List of RT-qPCR primers used in this study.

Name	Species	Applications	Forward	Reverse
*Β-Actin*	mouse	RT-qPCR	5′-GCCCTGAGGCTCTTTTCCAG-3′	5′-TGCCACAGGATTCCATACCC-3′
*Amh*	mouse	RT-qPCR	5′-TTTGGTGCTAACCGTGGACTTC-3′	5′-GAGCCAAATAGAAAGGCTTGCA-3′
*Cyp26a1*	mouse	RT-qPCR	5′-CTCGCACAAGCAGCGAAAG-3′	5′-GATCACGGGCACGTAGCACT-3′
*Cyp26b1*	mouse	RT-qPCR	5′-TGGACTGTGTCATCAAGGAGGT-3′	5′-GTCGTGAGTGTCTCGGATGCTA-3′
*Ddx4*	mouse	RT-qPCR	5′-GAAGAAATCCAGAGGTTGGC-3′	5′-GAAGGATCGTCTGTCTGAACA-3′
*HSD3b*	mouse	RT-qPCR	5′-TGGTGACAGGAGCAGGA-3′	5′-AGGAAGCTCACAGTTTCCA-3′
*Rara*	mouse	RT-qPCR	5′-TGTTTCGACGTGGGCATGT-3′	5′-TTTGTTTCGATCGTTTCGCA-3′
*Rarb*	mouse	RT-qPCR	5′-TTTAATCTGTGGAGACCGCCA-3′	5′-TTGTCTACTTTTGTTGGTTCCTCAAG-3′
*Rarg*	mouse	RT-qPCR	5′-GATGGATGACACCGAGACTGG-3′	5′-CCACAGATGAGGCAGATAGCAC-3′
*Stra8*	mouse	RT-qPCR	5′-TGAAGCTCAAAGCATCCTTCAA-3′	5′-CTAAGCTGTTGGGATTCCCATC-3′

**Table 2:** List of the antibodies used in the study.

Name	Company	Host Species	Mono/poly	Concentration	Applications
AMH	Santa Cruz	Goat	Poly	1/200	IHC
DDX4	Abcam	Mouse	Mono	1/200	IHC
DDX4	Abcam	Rabbit	Poly	1/200	IHC
RFP	Abcam	Rabbit	Poly	1/200	IHC
STRA8	Abcam	Rabbit	Poly	1/1000	IHC

## Supplementary Materials

The following are available online at https://www.mdpi.com/2218-273X/9/10/536/s1, Figure S1. Purified germ and somatic cell populations of fetal gonads express specific markers. Figure S2. Long-term inhibition of RA signaling prevents *Stra8* expression. Figure S3. Intracardiac injection and electroporation of plasmids allow the ectopic expression of proteins in fetal gonads. Figure S4. *Cyp26b1* or *Cyp26a1* overexpression equally inhibit *Rarb* expression. Figure S5. Ectopic expression of *Cyp26b1* or *Cyp26a1* decreases *Stra8* expression in fetal testes.
