# Supplementary material for: Divergent Roles of CYP26B1 and Endogenous Retinoic Acid in Mouse Fetal Gonads
Source: Biomolecules. 2019 Sep 26;9(10):536. doi: 10.3390/biom9100536 (PMC6843241; doi:10.3390/biom9100536)
Supplement: Supplementary file 1 [file biomolecules-09-00536-s001.pdf]

Supplementary Materials

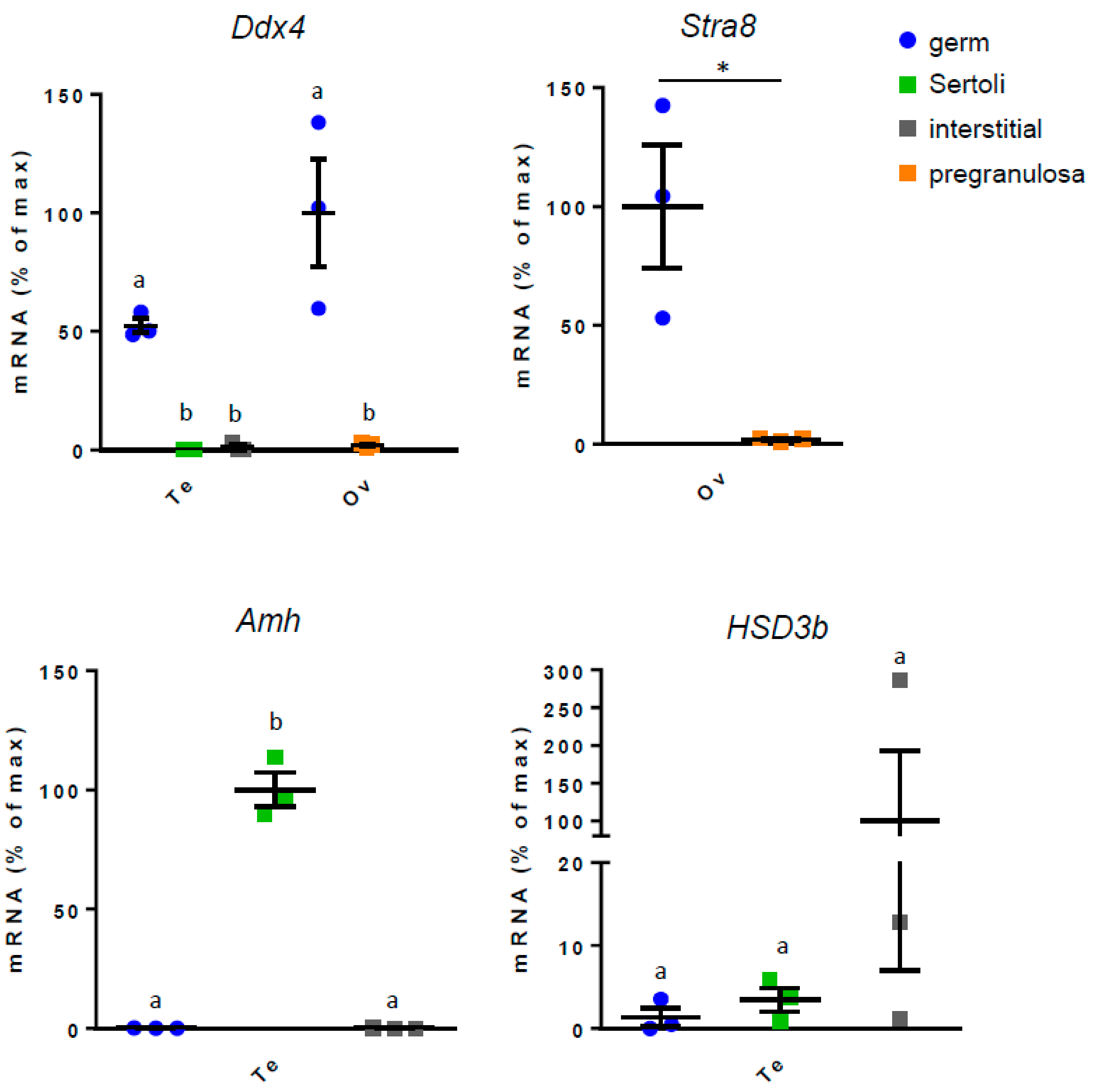

FigureS1. Purified germ and somatic cell populations of fetal gonads express specific markers. Analysis of mRNA expression of markers specific of the different purified population of gonad cells. *Ddx4*: germ cell marker; *Stra8*: pre-meiotic cell marker; *Amh*: Sertoli cell marker; *HSD3b*: Leydig cell marker.*Ddx4*, *Amh* and *HSD3b* values are normalised to the housekeeping gene *β-Actin*; *Stra8* values are normalised to the GC specific marker *Ddx4*. RNA levels are expressed as percentage of maximum (i.e.maximal values are defined as 100%). Different letters indicate significantly different data (multiple comparisons ANOVA). *p<0,05 (non-parametric-test) Mean ± SEM, n=3.

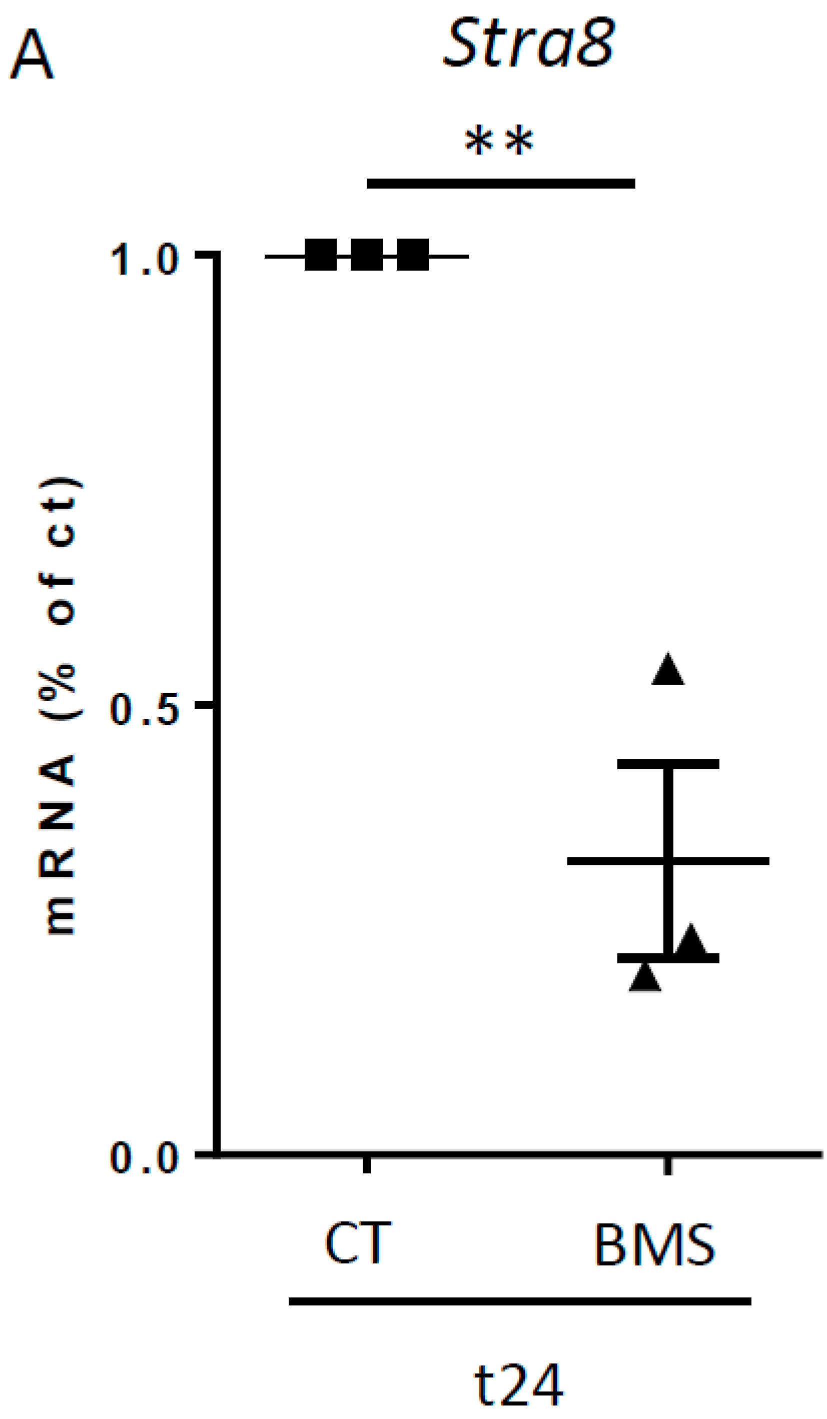

FigureS2. Long-term inhibition of RA signaling prevents *Stra8* expression. Analysis of mRNA expression of *Stra8* in 12.2 fetal ovaries cultured for 24 h (t24) in culture medium (CT) or in culture medium with 10-6M BMS 493. Values are normalised to the germ cell specific marker *Ddx4*. RNA levels are expressed as percentage of controls (i.e. t24 control values are defined as 100%). Mean ± SEM, n = 3.**p<0,01unpaired t-test.

A

	Fetus	Heart beating		Blue		E surface			
		Absent	Present	-	+	-	+	++	nd
E GFP	11	1	10	2	9	2	3	6	0
E B1	43	5	38	12	31	10	15	18	0
E A1	34	15	19	18	16	7	8	3	16
Tot	88	21	67	32	56	19	26	27	16

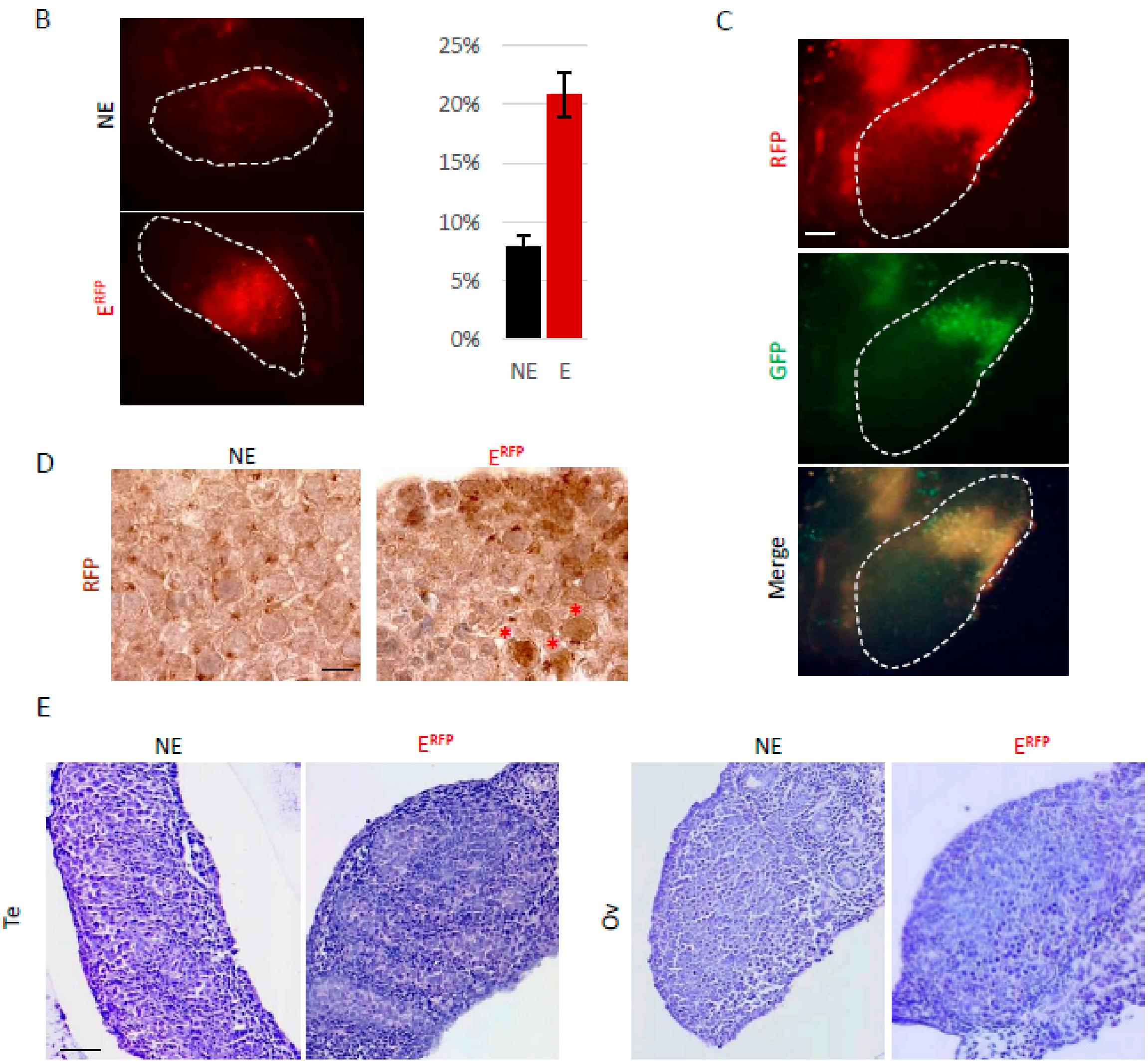

FigureS3. Intracardiac injection and electroporation of plasmids allow the ectopic expression of proteins in fetal gonads. **A**) Table summary of fetus electroporated with GFP (EGFP), Cyp26b1(EB1) or Cyp26a1(EA1). After intracardiac injection heart of embryos are still beating (Present) or not (Absent). After 30 min of incubation, intracardiac injected fetus are blue (+) or not (-). Electroporated (E) gonad surface is evaluated after 48 h of culture by macroscopic observation of RFP. We classified gonads into three categories depending on the intensity of the RFP:absent (-), between 5% and 15% (+) or > 15% (++) of red-fluorescenceinthegonad.**B**)Percentageofred-fluorescent surface of NE (n=5) or E (n=11) with

RFP electroporation. **C**) Macroscopic observation of an ovary co-electroporated with GFP and RFP: red and green fluorescence colocalises, indicating that plasmids are contransfected in the same cells. Scale bar: 200 µm **D**) Immunohistochemical analysis of RFP in sections of ovaries NE or E with RFP. Red stars indicate RFP-positive cells. Scale bar: 10 µm **E**) Testes (Te) and ovaries (Ov) NE or E with RFP stained with hematoxyline/eosine. Electroporation does not alter the morphology of fetal gonads. Scale bar: 50µm

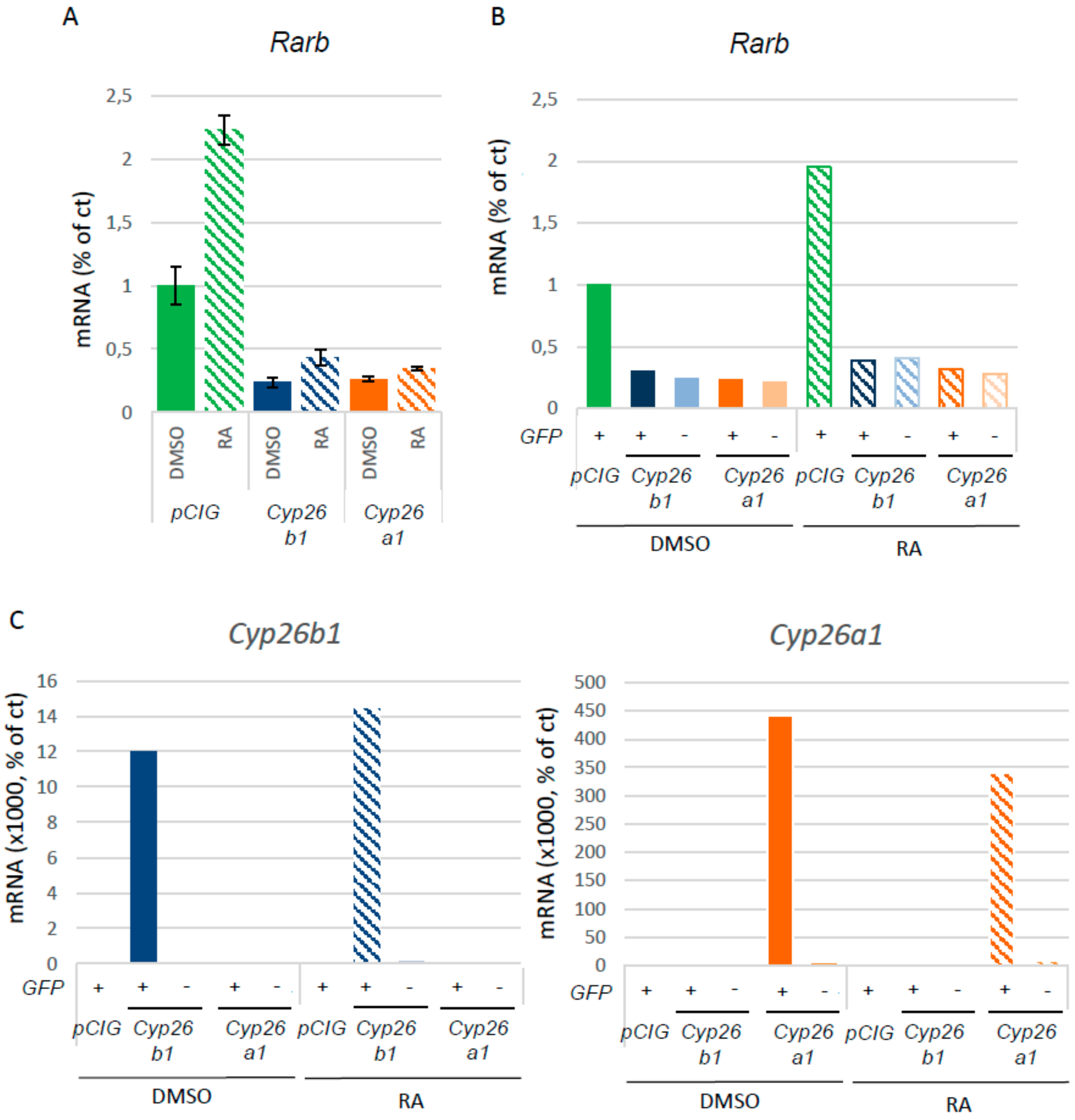

FigureS4. *Cyp26b1* or *Cyp26a1* over expression equally inhibits *Rarb2* expression. HEK-293 are transfected with pCIG, empty (GFP) or coding for Cyp26b1 or Cyp26a1 and cultured with DMSO (control) or 10-7M RA. **A**) After culture, mRNA expression level of the RA-target gene *Rarb2* is quantified by RT-qPCR. Values are normalised to the housekeeping gene *β-Actin*. mRNA levels are expressed as percentage of controls (i.e. control values are defined as 100%). Different letters indicate significantly different data (multiple comparisons ANOVA). Mean ± SEM, n = 5. **B**) and **C**) Transfected (GFP+) and non-transfected(GFP-)cellsofthesameplateareisolatedandanalysedindependentlybyRT-qPCR. **B**)

mRNA expression level of the RA-target gene *Rarb2*; **C**) mRNA expression level of *Cyp26b1* and *Cyp26a1* is quantified to verify the purification level of transfected and non-transfected cells. Values are normalised to the housekeeping gene *β-Actin*. mRNA levels are expressed as percentage of controls (i.e.control values are defined as 100%). n = 2

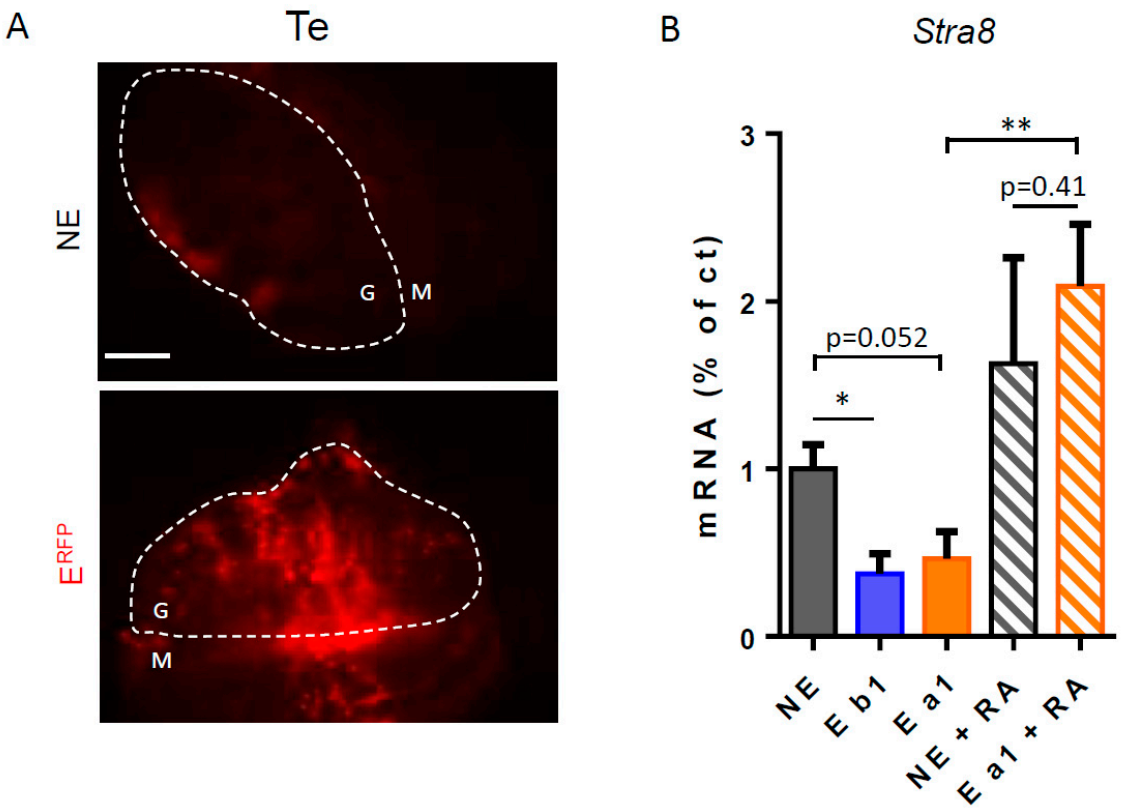

FigureS5. Ectopic expression of *Cyp26b1* or *Cyp26a1* decreases *Stra8* expression in fetal testes. **A**) RFP detection in testes (Te) NE or E with RFP. White dotted lines encercle gonads (G). Adjacent mesonephros (M) is indicated. Scale bar: 200 µm. **B**) 12.2 fetal testes are non-electroporated (NE) or electroporated with GFP (E GFP), *Cyp26b1* (E b1, n=5) or *Cyp26a1* (E a1, n=4). After 48 h of culture, mRNA expression level of *Stra8* is measured.Values are normalised to *Ddx4* (GC specific marker). The RA treatment of *Cyp26a1*-electroporated testes prevents the decrease of *Stra8* expression. a=0.052; *p<0.05; **p<0.01 (multiple comparisons ANOVA)
